# Jagn1 Is Induced in Response to ER Stress and Regulates Proinsulin Biosynthesis

**DOI:** 10.1371/journal.pone.0149177

**Published:** 2016-02-16

**Authors:** Courtney Nosak, Pamuditha N. Silva, Pietro Sollazzo, Kyung-Mee Moon, Tanya Odisho, Leonard J. Foster, Jonathan V. Rocheleau, Allen Volchuk

**Affiliations:** 1 Department of Physiology, University of Toronto, Toronto, Canada; 2 Institute for Biomaterials and Biomedical Engineering, University of Toronto, Toronto, Canada; 3 Department of Medical Biophysics, University of Toronto, Toronto, Canada; 4 Department of Biochemistry & Molecular Biology and Centre for High-Throughput Biology, University of British Columbia, Vancouver, Canada; 5 Keenan Research Centre of the Li Ka Shing Knowledge Institute, St. Michael’s Hospital, Toronto, Canada; Broad Institute of Harvard and MIT, UNITED STATES

## Abstract

The Jagn1 protein was indentified in a SILAC proteomic screen of proteins that are increased in insulinoma cells expressing a folding-deficient proinsulin. Jagn1 mRNA was detected in primary rodent islets and in insulinoma cell lines and the levels were increased in response to ER stress. The function of Jagn1 was assessed in insulinoma cells by both knock-down and overexpression approaches. Knock-down of Jagn1 caused an increase in glucose-stimulated insulin secretion resulting from an increase in proinsulin biosynthesis. In contrast, overexpression of Jagn1 in insulinoma cells resulted in reduced cellular proinsulin and insulin levels. Our results identify a novel role for Jagn1 in regulating proinsulin biosynthesis in pancreatic β-cells. Under ER stress conditions Jagn1 is induced which might contribute to reducing proinsulin biosynthesis, in part by helping to relieve the protein folding load in the ER in an effort to restore ER homeostasis.

## Introduction

Endoplasmic reticulum (ER) stress has been implicated in pancreatic β-cell dysfunction leading to the development of type 2 diabetes [1,2]. Several factors associated with obesity and type 2 diabetes can contribute to inducing ER stress in β-cells including elevated fatty acid levels, inflammatory cytokines, hyperglycemia and peripheral tissue insulin resistance, which forces the β-cell to produce and secrete excess insulin.

ER stress occurs when the protein folding load in the ER exceeds ER chaperone capacity or if the ER luminal environment is perturbed. Cells respond to ER stress by inducing the unfolded protein response (UPR), which aims to counteract ER stress by transiently inhibiting general protein translation followed by a transcriptional response that increases the mRNA levels and subsequently protein levels of numerous genes related to ER and secretory pathway function [3,4].

In order to understand how pancreatic β-cells respond to stress in the ER, we developed an insulinoma cell line model system that expresses the Akita mutant proinsulin (C96Y) that lacks a critical Cys residue and thus is unable to fold correctly in the ER[5]. Accumulation of misfolded proinsulin in the ER results in ER stress and the induction of the UPR [6]. Using this model cell line we performed a stable isotope labeling by amino acids in cell culture (SILAC) proteomic analysis to identify protein changes in response to mutant proinsulin expression. Here we show that one of the proteins identified in this screen, Jagn1, is induced in response to ER stress and modulates insulin biosynthesis in pancreatic β-cells.

## Materials and Methods

### Cell culture and mouse islet isolation

Rat INS-1 insulinoma cells were obtained from Dr. Claus Wollheim (University of Geneva) [7]. INS1 832/13 insulinoma cells were obtained from Dr. Chris Newgard (Duke University). INS-1 (Insulin 2 C96Y-GFP) cells (clone #4S2) were generated as described [8]. The cell lines were maintained as described in the respective references. Pancreatic islets were isolated from mouse pancreas tissue as reported previously [9]. High fat diet feeding was performed as described previously [9]. All animal procedures were approved and were performed in accordance with the animal use protocols at the Toronto Centre for Phenogenonics.

### SILAC proteomic analysis

INS-1 (Insulin 2 C96Y-GFP) cells were grown in RPMI media supplemented with dialyzed 10% FBS, 2 mM L-glutamine, 55 μM β-mercaptoethanol and 1% penicillin/streptomycin. Cells grown in Light media were further supplemented with 500 μM L-[^12^C]_6_-[^14^N]_2_-Lys and L-[^12^C]_6_-[^14^N]_4_-Arg, while cells grown in Heavy media were supplemented with 550 μM L-[^13^C]_6_-[^15^N]_2_-Lys and L-[^13^C]_6_-[^15^N]_4_-Arg as outlined previously [10]. Ins 2-C96Y cells were cultured in SILAC media for 14 days and trypsinized twice, allowing for at least 5 cell doubling events and nearly 100% heavy amino acid incorporation. Cells grown in heavy SILAC media were treated with 2 μg/mL doxycyline for 48 hours to induce expression of the Insulin 2(C96Y)-GFP construct.

Cells grown in both SILAC conditions were collected and lysed in 50 mM ammonium bicarbonate with 1% (w/v) sodium deoxycholate and incubated at 95°C for 5 min. Lysate protein concentration was quantified by the bicinchoninic acid (BCA) method. Light and Heavy labeled lysates were then mixed in a 1:1 protein ratio and subjected to LC-MS/MS as reported previously [11].

KEGG pathway enrichment was done using the online tool WebGestalt (http://bioinfo.vanderbilt.edu/webgestalt/) using protein IDs identified with significance B values < 0.05 in at least one experiment. KEGG enrichment p-values were calculated by hypergeometric test with BH correction.

### RNA isolation, reverse transcription PCR and real-time PCR analysis

Total RNA was isolated from rat INS-1 (Insulin 2 C96Y-GFP) cells or mouse islets using TRIzol (Invitrogen). To examine Jagn1 mRNA expression in rat and mouse cells and tissues, RT-PCR was performed using Jagn1 primers (rat forward: aaactgcagatggcgtctcgggcaggccca; rat reverse; aaagtcgactcatttacgtttctctcctgtgtgctgg; mouse forward: gcgtctcgggcaggcccgcga; mouse reverse; tttccgtttcttctcctgtgt). RT-PCR was performed using the Qiagen OneStep RT-PCR kit. The cDNA products were resolved using 1.5% agarose gels and stained using ethidium bromide.

Real-time PCR analysis was performed using the TaqMan Gene Expression system (Life Technologies) as described previously [12]. Gene-specific primers and control β-actin primers were obtained from Life Technologies: rat Jagn1 (Rn01421134_m1), mouse Jagn1 (Mm00471239_m1), Insulin 2 (Rn01774648_g1); rat β-actin (435291E). The XBP1 splicing assay was performed as described previously [12].

### Generation of Myc and GFP-tagged Jagn1 constructs

To generate an N-terminal Myc-tagged Jagn1 construct, an RT-PCR was first performed using total RNA isolated from rat INS-1 832/13 cells and Jagn1 primers containing 5’ Pst1 and 3’ Sal1 restriction sites. The cDNA was cloned into the pCMV-Tag3B plasmid (Agilent Tech.). The same protocol was used to generate the GFP-Jagn1 construct, which was cloned into the pEGFP-c1 vector (BD Biosciences). Plasmids were sequenced (AGCT Corp, Toronto) to confirm the Jagn1 insert sequence and that it was in frame.

### Immunofluorescence microscopy

Cells were grown in 12-well plates on glass coverslips and treatments were applied as described in the figure legends. The cells were then washed twice with PBS and fixed in 3% paraformaldehyde in PBS for 20 min at RT. Cells were then washed twice with PBS, followed by incubation with 100 mM glycine for 15 min. Cells were permeabilized with 0.1% Triton X-100 or 25 μg/ml digitonin in PBS for 15 min at RT. The cells were washed 3 times in PBS and blocked in 2% non-fat milk/ 2% BSA for 1 h. Primary antibody in blocking solution was added and samples were incubated for 1 h at RT. The cells were washed 3 times with PBS for 5 min each and incubated with secondary antibody in blocking solution for 1 h. The cells were washed 3 times for 5 min each and coverslipes were mounted onto glass slides using Fluoromount G mounting medium (EM Science). The following primary antibodies were used: Myc 9E10 (1:1000, Sigma M4439); Myc polyclonal (1:400, Cell Signaling 2272); CM1A10 (COPI) (1:500, obtained from Dr. J. Rothman, Yale University); insulin (1:300, Santa Cruz 9168); Protein Disulfide Isomerase (PDI) (1:500, Enzo Life Sciences Inc. SPA-890). Images were acquired with a Plan-Apochromat 63×/1.40 oil immersion objective of an LSM 710 confocal microscope (Carl Zeiss). Dual colour images of Myc-Jagn1and insulin (or PDI) were excited with 488 nm and 633 nm laser lines respectively. Images of Myc-tag stained cells permeabilized with digitonin or triton-X-100 were excited with the 488 nm laser line. Insulin and Myc-Jagn1 levels were analyzed by measuring the mean fluorescence intensity in cells expressing Myc-Jagn1 or untransfected cells (30–50 cells were analyzed from N = 3 experiments).

### Transient transfection and siRNA knock-down

INS-1 832/13 and HeLa cells were transiently transfected with N-terminal tagged Jagn1 constructs for immunofluorescence imaging and overexpression experiments. Cells were seeded in 12 well plates (500 000 cells/well) and the following day 4 μl of Lipofectamine reagent and 2 μg of plasmid DNA were each diluted in 100 μl Opti-MEM (Invitrogen) separately and incubated at RT for 5 min. The solutions were then combined and incubated at RT for 20 min. Subsequently, the Lipofectamine-DNA complexes (200 μl) were added to the media and the cells were incubated for 24–48 h prior to analysis.

To knock down Jagn1 in INS-1 832/13 cells, siRNA transfection using Lipofectamine RNAiMAX (Invitrogen) was performed according to the manufacturer’s protocol. Briefly, for each well of a 12 well plate, 12 pmol of either Jagn1 siRNA (Invitrogen) or Luciferase siRNA (non-specific control) was diluted in 200 μl Opti-MEM and incubated for 5 min at RT. Then 2 μl of Lipofectamine RNAiMAX reagent was added to each well and incubated for 30 min at RT. During this time, cells were trypsinized, resuspended in RPMI media and counted to obtain a final concentration of 500 000 cells/ml. Cells (1 ml/well) were then added to each well after the incubation period to achieve a final concentration of 10 nM siRNA. Plates were then incubated for 72 h. Three different Jagn1 siRNAs were tested and the one with the highest level of knock-down efficiency as monitored by qPCR was used for subsequent experiments.

### Insulin secretion analysis

To measure insulin secretion, INS-1 832/13 cells were seeded in 12-well plates (500 000 cells/well) and transfected with control siRNAs (against Luciferase) or Jagn1 siRNAs. The cells were then washed twice in Krebs-Ringer Bicarbonate Buffer (KBRH) supplemented with 0.1% BSA. The cells were then incubated in 1 ml/well KRBH/0.1% BSA for 1 h at 37 C. The cells were then incubated in KRBH/ 0.1% BSA containing 2.8 mM or 16.7 mM D-glucose for 1 h at 37 C. The plates were then placed on ice and 700 μl of media was collected and centrifuged at 500xg 4 C for 5 min in a microfuge. The supernatant was collected and stored at -80 C. The cells were lysed in RIPA buffer supplemented with protease inhibitors (Roche) and 0.5 mM PMSF. The protein concentration was measured using a BCA protein assay (Pierce Inc.). Insulin in the media and cell lysates was measured using a rat insulin RIA kit (Linco Inc.). Insulin (in ng) was adjusted for total cellular protein content in each condition. Assays were performed in duplicate for each condition and 3–4 independent experiments were analyzed. The results were normalized to the insulin values in the control siRNA condition and the results were expressed as a fold change.

### Proinsulin pulse-chase biosynthesis and transport assay

INS-1 832/13 cells were seeded in 12 well plates (500 000 cells/well) and transfected with control siRNAs (against luciferase) or Jagn1 siRNAs. Proinsulin biosynthesis was monitored as described previously [13]. Briefly, the cells were washed once with PBS and incubated in 0.5 ml labelling media consisting of ^35^[S]-methionine/ cysteine (100 μCi/0.5 ml, Perkin Elmer, NEG022T001MC) in methionine/ cysteine-free DMEM for 20 min at 37 C. The cells were then washed with PBS and incubated on ice for 20 min in lysis buffer (1% Triton X-100, 100 mM KCl, 2 mM EDTA, 20 mM HEPES, pH 7.3, supplemented with protease inhibitors (Roche) and 0.5 mM PMSF. Alternatively, following labelling, the cells were washed with warm PBS and incubated with regular DMEM serum-free media for 30 min at 37 C, then lysed (chase condition). Protein concentration in the lysates was measured and equal amounts of protein (~150 μg)/ condition were incubated with 5 μg of insulin antibody (Santa Cruz) overnight at 4C with rotation. The next day lysates were incubated with Protein A Dynabeads (25 μl) for 3 h at 4 C with rotation. The magnetic beads were washed 3 times with lysis buffer then once with PBS/ 0.1% TX-100. The beads were then incubated with 2X NuPage sample buffer supplemented with 10% β-mercaptoethanol and boiled for 5 min. The sample buffer lysates were resolved on 4–12% Nupage gels, which were subsequently stained with Coomassie blue, washed extensively and dried at 52 C for 3 h. Once dried, the gel was exposed to a Phosphor screen (Amersham Biosciences) and processed using a PhosphorImager (Storm 840, Molecular Dynamics). Three independent experiments were performed and proinsulin band densitometry was quantified using ImageQuant software.

### Western blot analysis

INS-1 832/13 cells were lysed as described previously. Proteins were resolved using 10% SDS-PAGE gels or 4–12% NuPAGE gels (Invitrogen) and transferred to nitrocellulose membranes as described [12]. Antibodies used: Myc 9E10 (1:1000, Sigma-Aldrich M4439); γ-tubulin (1:1000, Sigma-Aldrich T6557); insulin (1:100, Santa Cruz 9168); GRP78 (1:1000, BD Bioscience 610978).

### Statistical analysis

Results are presented as mean +/-SE. Statistical significance between two experimental conditions was analyzed using a two sample t-test assuming equal variance. p<0.05 was considered significant.

## Results

**Fig 1:**
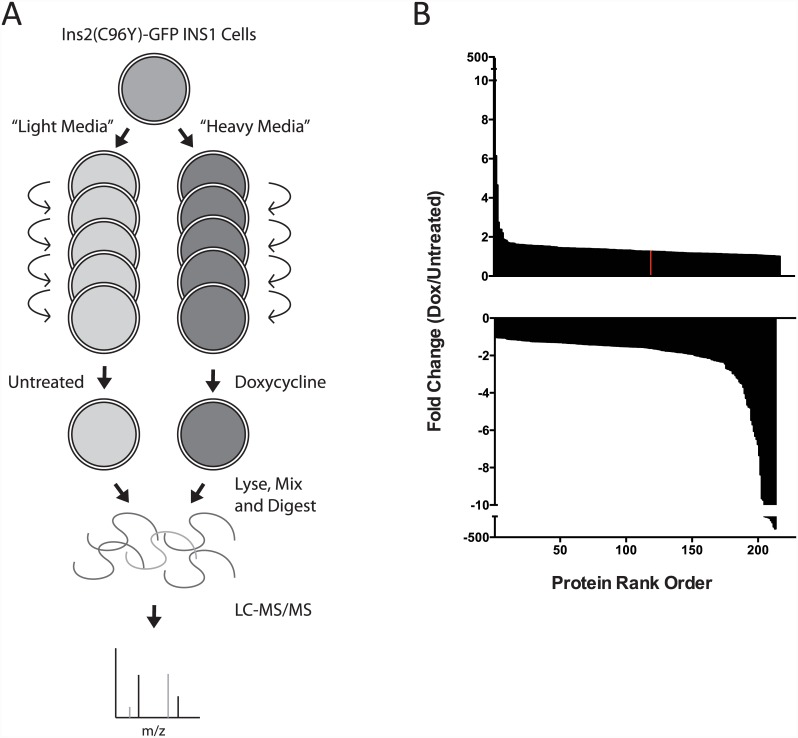
Proteomic analysis of the mutant proinsulin-induced ER stress response. (A) Rat INS-1 insulinoma cells harboring a doxycycline (dox)—inducible Ins2(C96Y)-GFP transgene were split into “heavy” media containing heavy isotope Lysine and Arginine and treated with doxycycline (dox) for 48 hours. Total protein was then mixed with mock treated cells grown in “light” media containing normal isotopic Lysine and Arginine. Following tryptic digestion, relative peptide abundance was analyzed by LC-MS/MS. (B) Protein expression between dox and mock treated cells was calculated from identified peptides that met statistical significance in at least one of four experiments and plotted for increased (upper) and decreased (lower) fold change. The Jagn1 protein is indicated in red.

Expression of a mutant proinsulin C96Y-GFP fusion protein causes ER stress and the induction of the UPR in a cultured insulinoma cell line model system [6]. To identify proteins whose levels change in response to mutant proinsulin production we performed a SILAC proteomic analysis as outlined in Fig 1A. Overall, the changes observed were generally small with relatively few proteins increased or decreased >1.5 fold, and there was considerable variability between experiments. Proteins that increased or decreased in doxycyline-treated cells (mutant insulin expression) that met statistical significance (sigB <0.05) in a least one of four independent experiments are graphed in Fig 1B (and listed in S1 File). Approximately 200 proteins were increased and despite the variability and small fold changes, as expected, ER proteins represented a significant proportion of those increased (see S2 File). The global SILAC analysis produced variable results likely due to the fact that in this model system protein changes are small, and thus the high variability. This reflects the biology of the system, which unlike pharmacology produces rather modest changes at the protein level. Looking at the proteins increased in response to mutant proinsulin expression many were expected proteins with clear biological functions and many are well known to be increased in response to stress in the endoplasmic reticulum. Jagn1 was chosen as a validation protein and for further functional analysis because it appears to be an ER-localized protein with limited functional information. Jagn1 was first identified in Drosophila egg cells and shown to be required for normal protein secretion during egg cell development [14].

**Fig 2:**
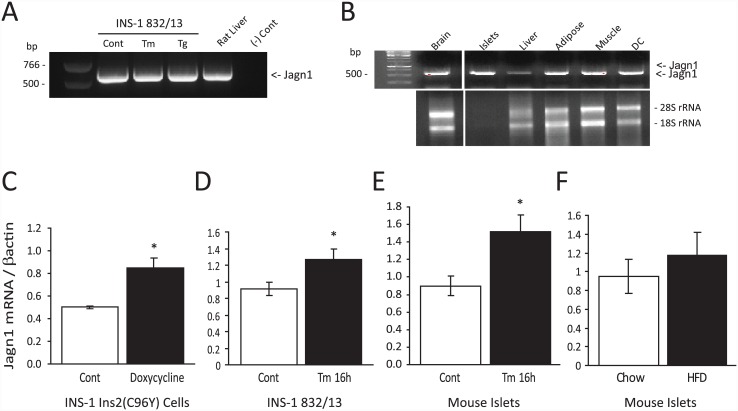
Jagn1 is expressed in insulinoma cells and islets and is induced by ER stress. (A) RT-PCR was performed to examine Jagn1 expression. Total RNA was isolated from control rat INS-1 832/13 insulinoma cells or cells treated with 2 μg/ml tunicamycin (Tm) for 16 h, 1 μM thapsigargin (Tg) for 6 h or from rat liver tissue. The (-) Control lane lacked one of the primers in the PCR reaction. (B) Total RNA was isolated from the indicated tissues and RT-PCR analysis was performed to detect Jagn1 expression. DC; dendritic cells. Lower panel shows rRNA in the total RNA detected by ethidium bromide (1 μg loaded for all samples except 100 ng islet RNA was loaded). (C-F) qPCR analysis for Jagn1 expression. (C) INS-1 insulin 2 (C96Y) cells treated or not with doxycycline (2 μg/ml) to induce mutant proinsulin expression for 24 h prior to total RNA isolation. (D) INS-1 832/13 cells treated with 2 μg/ml tunicamycin for 16 h. (E) Isolated mouse islets treated or not with tunicamycin as in D. (F) Islets isolated from mice fed a normal chow diet or a 45% high fat diet (HFD) for 12 weeks. In all cases results are from a minimum of N = 3 experiments. *p<0.05.

By RT-PCR we detected Jagn1 mRNA expression in INS-1 832/13 cells with no discernable difference in control or ER stressed cells (Fig 2A). Jagn1 mRNA was also detected in various tissues examined (brain, liver, adipose, muscle and dendritic cells), but appears to be particularly enriched in pancreatic islets (Fig 2B). To determine if Jagn1 is an ER stress response gene, we performed real-time PCR analysis and indeed, in line with the SILAC proteomic results, Jagn1 mRNA was significantly increased in insulinoma cells expressing mutant proinsulin (Fig 2C). Jagn1 mRNA was also increased in response to tunicamycin, an inhibitor of N-linked glycosylation known to induce ER stress, in insulinoma cells (Fig 2D) and in isolated primary mouse islets (Fig 2E). A chronic 20-week HFD has been shown to cause mild ER stress in mouse islets [9], but Jagn1 levels were not significantly changed in islets from HFD mice compared to chow fed mice (Fig 2F). These data suggest that Jagn1 expression is elevated in beta-cells by moderate to severe ER stress.

**Fig 3:**
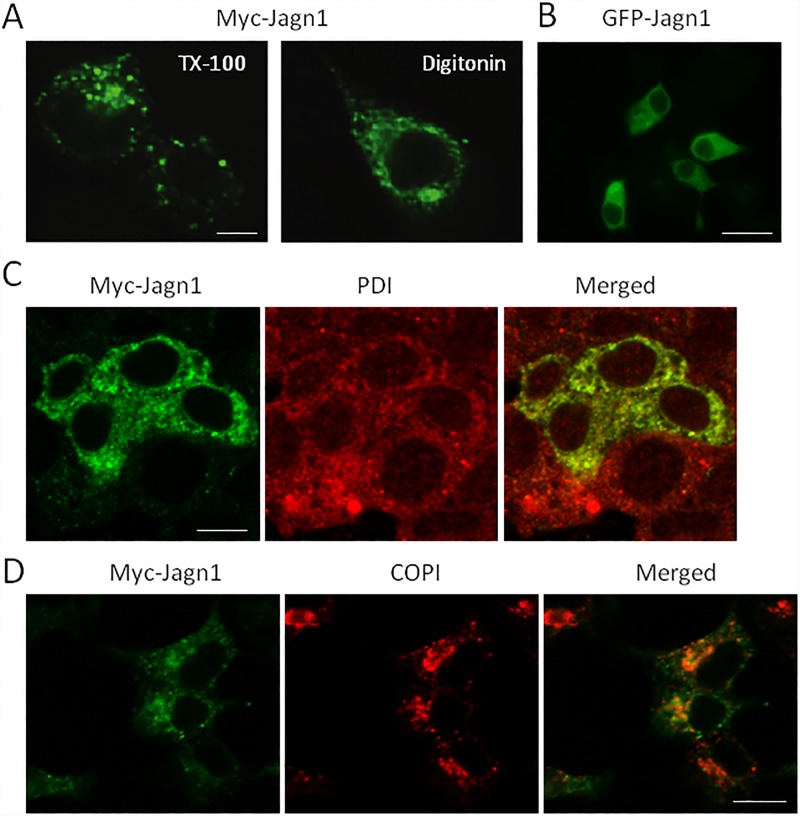
Jagn1 is primarily an ER localized membrane protein. (A) N-terminally Myc-tagged Jagn1 was expressed in HeLa cells. Cells were fixed in paraformladehyde and permeabilized with either TX-100 or digitonin and immunostained for the Myc-tag. Representative confocal images. Scale bar: 10 μm. (B) N-terminal GFP-tagged Jagn1 expressed in INS1 832/13. Scale bar: 10 μm. (C) Myc-tagged Jagn1 was expressed in βTC3 cells, fixed, permeabilized with TX-100 and immunostained for the Myc-tag and protein disulfide isomerise (PDI). Scale bar: 10 μm. (D) Myc-tagged Jagn1 was expressed in INS-1 832/13 cells, fixed, permeabilized with digitonin and immunostained for the Myc-tag and COPI coatomer. Scale bar: 10 μm.

Jagn1 was first discovered in *Drosophila* oocytes in a screen for genes required for egg development and shown to be an ER-localized membrane protein with 4 predicted transmembrane domains and an ER retention motif at its C-terminus [14]. To examine Jagn1 localization in insulinoma cells, we expressed Myc or GFP-tagged Jagn1 at the N-terminus, followed by immunofluorescence microscopy. Using TX-100 to permeabilize the cells resulted in a punctuate localization pattern (Fig 3A). In contrast, permeabilization with digitonin, which permeabilizes the plasma membrane but not the ER membrane, resulted in a reticular localization pattern typical of the ER. A similar localization pattern was observed with GFP-tagged Jagn1 (Fig 3B). To verify ER localization we transfected Jagn1 into the larger βTC3 cell line and observed significant co-localization with PDI, a luminal ER protein (Fig 3C). With Myc-tagged Jagn1 and less so with GFP-tagged Jagn1, in confocal sections through the middle of the cell some co-localization was also observed with COPI coatomer, which is used to generate retrograde targeted vesicles from the ER-Golgi intermediate compartment (ERGIC) and *cis*-Golgi [15,16] (Fig 3D). Thus, Jagn1 appears to be mainly localized to the ER and any that escapes the ER is likely efficiently returned via retrograde COPI vesicles that form from the ERGIC and/or cis-Golgi.

**Fig 4:**
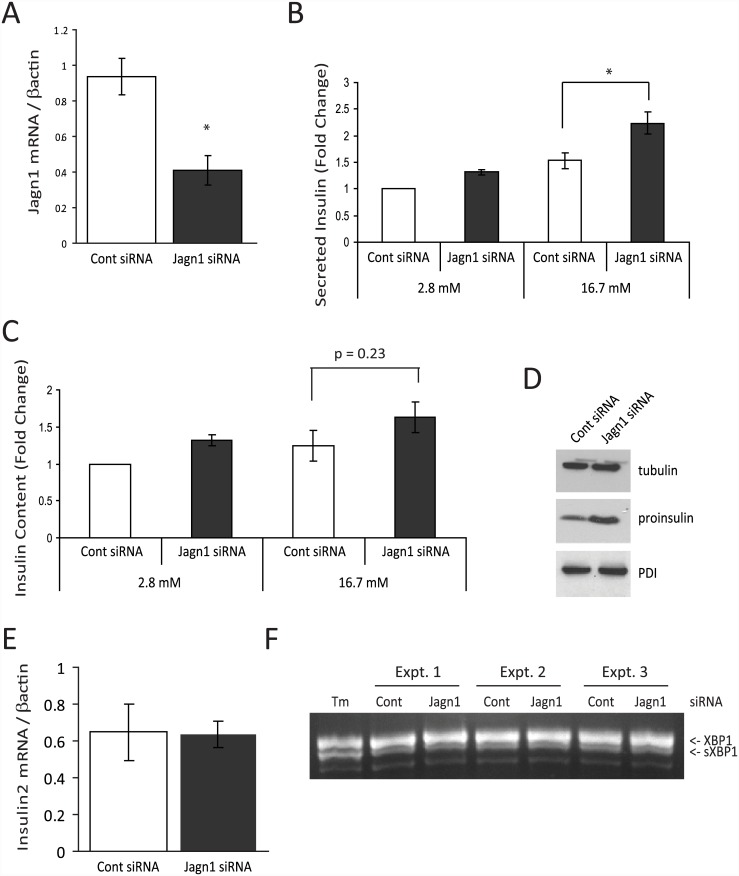
Knock-down of Jagn1 enhances insulin secretion and increases insulin content. (A) INS-1 832/13 cells were transfected with Jagn1 siRNA (10 nM) or control siRNA (directed to firefly luciferase) for 72 h. Total RNA was isolated and qPCR analysis was performed for Jagn1 expression (N = 3, *p<0.05). (B) INS-1 832/13 cells were transfected with Jagn1 siRNA or control siRNA as in (A). The cells were then treated with basal glucose (2.8 mM) or stimulatory glucose (16.7 mM) for 1 h. Insulin in the media was measured by RIA. Results are expressed as secreted insulin normalized to control siRNA transfected cells at basal glucose (N = 4, *p<0.05). (C) Cellular insulin content measured by RIA in the experiment in (B). (D) Cells were transfected with control or Jagn1 siRNA as in (A), cell lysates were prepared and western blot analysis was performed to detect proinsulin or tubulin (loading control protein). Representative of N = 2 experiments. (E) qPCR analysis of insulin2 gene expression in control or Jagn1 siRNA treated cells (10 nM for 72 h) (N = 3). (F) INS-1 832/13 cells were transfected with control siRNA or Jagn1 siRNA for 72 h. Total RNA was then isolated and levels of unspliced and spliced XBP1 mRNA were detected by RT-PCR. Cells treated with tunicamycin (2 μg/ml) (Tm) for 16 h was used as a positive control. Results from three independent experiments is shown (Expt.1–3).

As Jagn1 is localized to the ER and is induced in response to ER stress we examined if it might be required for insulin biosynthesis or secretion. Using siRNA we knocked-down Jagn1 mRNA by about 60% in insulinoma cells (Fig 4A). We were unable to access protein levels due to lack of a useful antibody. In control or Jagn1 siRNA-transfected cells we monitored insulin secretion after 1 h at both basal (2.8 mM) and stimulatory (16.7 mM) glucose concentrations. Under stimulatory conditions, insulin secretion into the media was significantly enhanced in cells depleted of Jagn1 (Fig 4B). The increased secretion could possibly be due to a general increase in cellular insulin content in Jagn1 depleted cells, which tended to be higher (Fig 4C). Jagn1 knock-down cells also have higher proinsulin content (Fig 4D), with no change in insulin 2 mRNA (Fig 4E), compared to control siRNA-transfected cells.

**Fig 5:**
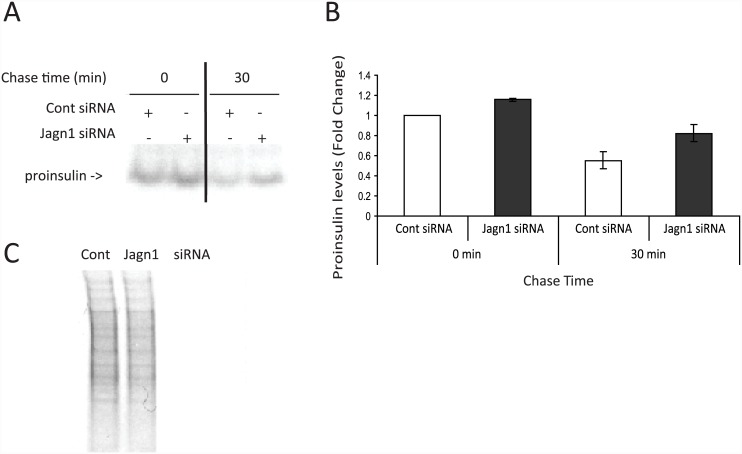
Jagn1 knock-down enhances proinsulin biosynthesis in INS-1 832/13 cells. (A) INS-1 832/13 cells were transfected with Jagn1 siRNA (10 nM) or control siRNA (directed to firefly luciferase) for 72 h. The cells were then washed in Met and Cys-free media and incubated in media containing ^35^[S]-Met/Cys for 20 min. The cells were then either lysed (time 0) or the cells were washed in PBS and incubated in regular media for 30 min (chase), prior to cell lysis. Cell lysates were immunoprecipitated with anti-insulin antibody, the immunoprecipitates were resolved by NuPage gels (Invitrogen) and newly synthesized proinsulin was detected by PhosphorImager analysis (result is representative of N = 3 experiments). (B) The proinsulin band was quantified by densitometry and expressed relative to levels in control siRNA treated cells at time 0. (C) Analysis of general protein translation after 20 min of ^35^[S]-Met/Cys labelling in control and Jagn1 knock-down cells.

The higher proinsulin and likely insulin content suggests that Jagn1 may affect proinsulin biosynthesis with an inhibitory role. To directly assess proinsulin biosynthesis we knocked down Jagn1 then labelled the cells with ^35^[S]Meth/Cys followed by proinsulin immunoprecipitation (Fig 5). Even after 20 min of labelling Jagn1 knock-down cells tended to have higher levels of radiolabelled proinsulin (Fig 5A), although the effect was small and did not reach significance (Fig 5B). Following a 30 min chase in regular medium proinsulin levels decreased as proinsulin leaves the ER and is processed in the Golgi. The rate of proinsulin exit was similar in control vs. Jagn1 siRNA transfected cells indicating that ER export is not significantly affected. Analysis of radiolabelled proteins between control and Jagn1 knock-down cells did not reveal major differences, suggesting that Jagn1 does not affect general protein translation (Fig 5C). Thus, Jagn1 knock-down cells had a trend towards higher levels of proinsulin biosynthesis (Fig 5), consistent with the higher steady-state levels of proinsulin observed by western blotting (Fig 4D).

**Fig 6:**
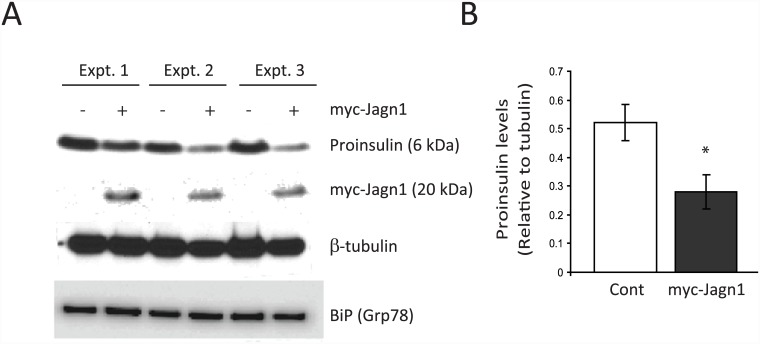
Overexpression of Jagn1 decreases proinsulin levels. (A) INS-1 832/13 cells were transfected or not with Myc-Jagn1 plasmid. After 24 h the cells were lysed and immunoblotted for the indicated proteins. Results from three independent experiments is shown (Expt.1–3). (B) Cellular proinsulin levels were quantified.

**Fig 7:**
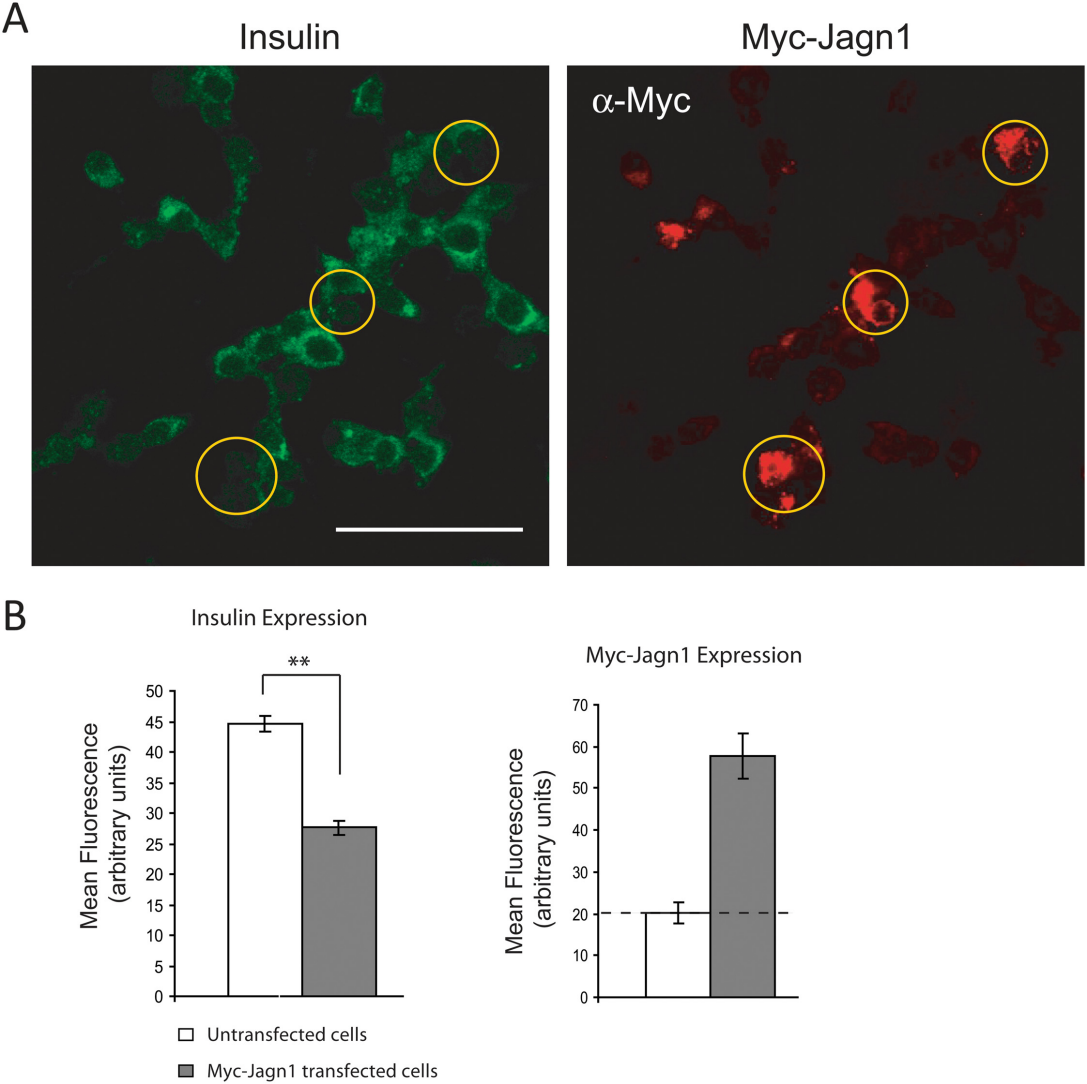
Overexpression of Jagn1 decreases steady-state insulin levels. (A) INS-1 832/13 cells were transfected with Myc-Jagn1 plasmid. After 24 h the cells were fixed and immunostained for insulin (green) and Myc-Jagn1 (red) and imaged by confocal microscopy. Scale bar: 50 μM. (B) Cellular insulin levels were quantified by measuring fluorescence intensity expressed as mean fluorescence in arbitrary units.

Given that knock-down of Jagn1 increases proinsulin biosynthesis, we tested whether overexpression might have the opposite effect. Indeed, overexpression of Myc-tagged Jagn1 resulted in significantly reduced steady-state levels of proinsulin compared to control cells (Fig 6). This did not affect the levels of other ER-localized proteins such as BiP (GRP78) (Fig 6). We also monitored steady-state cellular insulin levels by immunofluorescence microscopy. INS1 832/13 cells were transfected with Myc-Jagn1 and the cells were co-immunostained with anti-Myc and anti-insulin antibodies. In cells transfected with Myc-Jagn1 the insulin levels were lower compared to neighbouring untransfected cells (Fig 7). Thus, Jagn1 overexpression reduces cellular proinsulin and insulin levels.

## Discussion

In a SILAC proteomic screen we identified Jagn1 as a protein whose levels were increased in an insulinoma cell model of misfolded proinsulin induced ER stress. We confirmed that Jagn1 mRNA is detected in rodent islets and insulinoma cells, and that expression of this gene appears to be ubiquitous. We also validated that Jagn1 mRNA is increased in insulinoma cells by conditions that cause moderate to severe ER stress including production of misfolded proinsulin and tunicamycin treatment. Thus, Jagn1 mRNA and likely protein levels are increased in cells undergoing ER stress. This result is consistent with microarray analysis of tunicamycin-treated mouse embryonic fibroblasts (MEFs) from control and CHOP knock-out mice, indicating the involvement of the CHOP pathway in Jagn1 induction [17].

To study the potential function of Jagn1 in pancreatic β-cells we used both knock-down and overexpression approaches. Knock-down of Jagn1 by ~60% at the mRNA level resulted in enhanced insulin secretion under glucose-stimulated conditions, which is most likely accounted for by an increase in proinsulin translation in Jagn1 knock-down cells. The effects observed, although significant, were generally small. However, this may be due to suboptimal knock-down efficiency as we were not able to examine endogenous Jagn1 protein levels in the knock-down experiments. Importantly however, overexpression of Myc-tagged Jagn1 in insulinoma cells caused the opposite effect; i.e. a reduction in steady-state proinsulin and insulin levels.

Collectively these results indicate that Jagn1 is an ER stress inducible gene that in pancreatic β-cells can modulate proinsulin biosynthesis. Thus, in pancreatic β-cells undergoing ER stress Jagn1 may contribute to putting a break on proinsulin translation, which would be a beneficial effect under such conditions. Once ER homeostasis is restored Jagn1 levels would reduce to basal levels and thereby restoring normal proinsulin translation. This mechanism probably works after the PERK/eIF2α system that reduces global translation rates as part of the initial ER stress response. The PERK/eIF2α system is activated early to acutely reduce global translation to reduce the protein folding load on the ER as mRNAs for ER and secretory pathway genes accumulate. Once translation resumes Jagn1 will be translated and depending on its levels will dampen insulin translation. This may act to fine tune insulin production based on the protein folding capacity of the ER.

How might Jagn1 affect proinsulin translation in insulinoma cells? This question remains unanswered. However, other than COPI coatomer, which binds the C-terminal KKXX domain in Jagn1, the main putative interacting proteins identified in a biochemical proteomic analysis [18] and in a Membrane Yeast Two-Hybrid screen (our unpublished results) are ribosomal proteins. Thus, Jagn1 may modulate translation of abundant mRNAs in distinct cell types including proinsulin in β-cells by sequestering certain ribosomal subunits such as L13a. Indeed, the L13a ribosomal protein mediates translational silencing of certain inflammatory proteins in macrophage cells and depletion of L13a abrogates the translational repression of these proteins [19]. A similar mechanism could explain the enhanced proinsulin translation rate in Jagn1 knock-down cells and the reduced rate in cells overexpressing Jagn1. Additional studies are required to determine the exact mechanism by which Jagn1 modulates proinsulin translation.

Jagn1 is an ER localized protein with 4 putative membrane spanning domains with both the N and C-terminus in the cytosol. In *Drosophila* embryos it plays an essential role in organizing the ER and secretion of certain proteins required during egg development [14]. More recently, the Jagn1 gene has been shown to be mutated in patients with a rare form of neutropenia and shown to play a role in the secretory pathway, since certain cell surface receptors such as the G-CSF receptor are reduced in patient neutrophil cells [18]. It is notable that these individuals often have other abnormalities in addition to neutropenia including some with abnormal pancreatic function, although it does not appear these young individuals have major metabolic abnormalities [18]. The Jagn1 gene appears to be essential in mice [20] and thus will require tissue-specific deletion to examine its function *in vivo*. It remains to be determined if Jagn1 plays a significant role in regulating proinsulin translation in pancreatic β-cells under physiological or pathological ER stress conditions *in vivo*.

## Supporting Information

S1 File Results from the SILAC analysis. List of proteins identified by SILAC proteomic analysis with a significance B value less than 0.05 in at least one of four independent experiments with their corresponding fold change values relative to uninduced cells.
(CSV)

S2 File KEGG pathway analysis of proteins increased in response to mutant proinsulin production. List of top 10 enriched KEGG terms identified from proteins whose expression changes are greater than 1.3 fold and have a significance B value less than 0.05 when induced with ER stress caused by mutant proinsulin production. For each KEGG term the number of genes, Entrez gene IDs and BH corrected p-values are shown.
(XLSX)

## References

[1] Eizirik DL, Cardozo AK, Cnop M (2008) The role for endoplasmic reticulum stress in diabetes mellitus. Endocr Rev 29: 42–61. 18048764 10.1210/er.2007-0015

[2] Eizirik DL, Miani M, Cardozo AK (2013) Signaling danger: endoplasmic reticulum stress and the unfolded protein response in pancreatic islet inflammation. Diabetologia 56: 234–241. 10.1007/s00125-012-2762-3 23132339

[3] Ron D, Walter P (2007) Signal integration in the endoplasmic reticulum unfolded protein response. Nat Rev Mol Cell Biol 8: 519–529. 17565364 10.1038/nrm2199

[4] Walter P, Ron D (2011) The unfolded protein response: from stress pathway to homeostatic regulation. Science 334: 1081–1086. 10.1126/science.1209038 22116877

[5] Wang J, Takeuchi T, Tanaka S, Kubo SK, Kayo T, Lu D, et al (1999) A mutation in the insulin 2 gene induces diabetes with severe pancreatic beta-cell dysfunction in the Mody mouse. The Journal of clinical investigation 103: 27–37. 9884331 10.1172/JCI4431 PMC407861

[6] Hartley T, Siva M, Lai E, Teodoro T, Zhang L, Volchuk A (2010) Endoplasmic reticulum stress response in an INS-1 pancreatic beta-cell line with inducible expression of a folding-deficient proinsulin. BMC cell biology 11: 59 10.1186/1471-2121-11-59 20659334 PMC2921384

[7] Asfari M, Janjic D, Meda P, Li G, Halban PA, Wollheim CB (1992) Establishment of 2-mercaptoethanol-dependent differentiated insulin-secreting cell lines. Endocrinology 130: 167–178. 1370150 10.1210/endo.130.1.1370150

[8] Hohmeier HE, Mulder H, Chen G, Henkel-Rieger R, Prentki M, Newgard CB (2000) Isolation of INS-1-derived cell lines with robust ATP-sensitive K+ channel-dependent and -independent glucose-stimulated insulin secretion. Diabetes 49: 424–430. 10868964 10.2337/diabetes.49.3.424

[9] Teodoro-Morrison T, Schuiki I, Zhang L, Belsham DD, Volchuk A (2013) GRP78 overproduction in pancreatic beta cells protects against high-fat-diet-induced diabetes in mice. Diabetologia 56: 1057–1067. 10.1007/s00125-013-2855-7 23475366

[10] Ong SE, Foster LJ, Mann M (2003) Mass spectrometric-based approaches in quantitative proteomics. Methods: 124–130. 12606218 10.1016/s1046-2023(02)00303-1

[11] Auweter SD, Bhavsar AP, de Hoog C, Li Y, Chan YA, van der Heijden J, et al (2011) Quantitative mass spectrometry catalogues Salmonella pathogenicity island-2 effectors and identifies their cognate host binding partners. J Biol Chem 286: 24023–24035. 10.1074/jbc.M111.224600 21566117 PMC3129184

[12] Zhang L, Lai E, Teodoro T, Volchuk A (2009) GRP78, but Not Protein-disulfide Isomerase, Partially Reverses Hyperglycemia-induced Inhibition of Insulin Synthesis and Secretion in Pancreatic {beta}-Cells. The Journal of biological chemistry 284: 5289–5298. 10.1074/jbc.M805477200 19103594

[13] Zhang L, Volchuk A (2010) p24 family type 1 transmembrane proteins are required for insulin biosynthesis and secretion in pancreatic beta-cells. FEBS Lett 584: 2298–2304. 10.1016/j.febslet.2010.03.041 20353786

[14] Lee S, Cooley L (2007) Jagunal is required for reorganizing the endoplasmic reticulum during Drosophila oogenesis. J Cell Biol 176: 941–952. 17389229 10.1083/jcb.200701048 PMC2064080

[15] Brandizzi F, Barlowe C (2013) Organization of the ER-Golgi interface for membrane traffic control. Nat Rev Mol Cell Biol 14: 382–392. 10.1038/nrm3588 23698585 PMC4064004

[16] Lee MC, Miller EA, Goldberg J, Orci L, Schekman R (2004) Bi-directional protein transport between the ER and Golgi. Annu Rev Cell Dev Biol 20: 87–123. 15473836 10.1146/annurev.cellbio.20.010403.105307

[17] Marciniak SJ, Yun CY, Oyadomari S, Novoa I, Zhang Y, Jungreis R, et al (2004) CHOP induces death by promoting protein synthesis and oxidation in the stressed endoplasmic reticulum. Genes & development 18: 3066–3077. 15601821 10.1101/gad.1250704 PMC535917

[18] Boztug K, Jarvinen PM, Salzer E, Racek T, Monch S, Garncarz W, et al (2014) JAGN1 deficiency cuases aberrant myeloid cell homeostasis and congenital neutropenia. Nat Genet 46: 1021–1027. 10.1038/ng.3069 25129144 PMC4829076

[19] Basu A, Poddar D, Robinet P, Smith JD, Febbraio M, Baldwin WM III, et al (2014) Ribosomal protein L13a deficiency in macrophages promotes atherosclerosis by limiting translation control-dependent retardation of inflammation. Arterioscler Thromb Vasc Biol 34: 533–542. 10.1161/ATVBAHA.113.302573 24436370 PMC3954853

[20] Wirnsberger G, Zwolanek F, Stadlmann J, Tortola L, Liu SW, Perlot T, et al (2014) Jagunal homolog 1 is a critical regulator of neutrophil function in fungal host defense. Nat Genet 46: 1028–1033. 10.1038/ng.3070 25129145 PMC6245568

